# Dietary reversal of neuropathy in a murine model of prediabetes and metabolic syndrome

**DOI:** 10.1242/dmm.028530

**Published:** 2017-06-01

**Authors:** Lucy M. Hinder, Phillipe D. O'Brien, John M. Hayes, Carey Backus, Andrew P. Solway, Catrina Sims-Robinson, Eva L. Feldman

**Affiliations:** 1 Department of Neurology, University of Michigan, Ann Arbor, MI 48109, USA; 2 Department of Neurology, Medical University of South Carolina, Charleston, SC 29425, USA

**Keywords:** Diabetic complications, High-fat diet, Obesity, Impaired glucose tolerance, Dietary intervention, Strain comparison

## Abstract

Patients with metabolic syndrome, which is defined as obesity, dyslipidemia, hypertension and impaired glucose tolerance (IGT), can develop the same macro- and microvascular complications as patients with type 2 diabetes, including peripheral neuropathy. In type 2 diabetes, glycemic control has little effect on the development and progression of peripheral neuropathy, suggesting that other metabolic syndrome components may contribute to the presence of neuropathy. A parallel phenomenon is observed in patients with prediabetes and metabolic syndrome, where improvement in weight and dyslipidemia more closely correlates with restoration of nerve function than improvement in glycemic status. The goal of the current study was to develop a murine model that resembles the human condition. We examined longitudinal parameters of metabolic syndrome and neuropathy development in six mouse strains/genotypes (BKS-*wt*, BKS-*Lepr^db/+^*, B6-*wt*, B6-*Lepr^db/+^*, BTBR-*wt*, and BTBR*-Lep^ob/+^*) fed a 54% high-fat diet (HFD; from lard). All mice fed a HFD developed large-fiber neuropathy and IGT. Changes appeared early and consistently in B6-*wt* mice, and paralleled the onset of neuropathy. At 36 weeks, B6-*wt* mice displayed all components of the metabolic syndrome, including obesity, IGT, hyperinsulinemia, dyslipidemia and oxidized low density lipoproteins (oxLDLs). Dietary reversal, whereby B6-*wt* mice fed a HFD from 4-20 weeks of age were switched to standard chow for 4 weeks, completely normalized neuropathy, promoted weight loss, improved insulin sensitivity, and restored LDL cholesterol and oxLDL by 50% compared with levels in HFD control mice. This dietary reversal model provides the basis for mechanistic studies investigating peripheral nerve damage in the setting of metabolic syndrome, and ultimately the development of mechanism-based therapies for neuropathy.

## Abstract (precis)

**Summary:** A mouse model of metabolic syndrome can be used to investigate peripheral nerve damage, enabling the development of mechanism-based therapies for neuropathy.

## INTRODUCTION

The number of individuals with prediabetes and type 2 diabetes mellitus (T2DM) is increasing in parallel with the rise in obesity. In the United States, 35% of adults, and 17% of children and young adults, are obese and at risk of developing prediabetes (Ogden et al., 2014). Prediabetes is characterized by impaired glucose tolerance, and often develops into T2DM. Both prediabetes and T2DM are components of the metabolic syndrome, whose other risk factors include obesity, dyslipidemia and hypertension. Prediabetic patients largely develop the same macro- and microvascular complications as patients with T2DM, including neuropathy (Callaghan et al., 2012b,b). Early in the course of prediabetic neuropathy, patients frequently report burning pain and allodynia. The neurological examination in turn reveals a distal to proximal loss of thermal or pinprick sensation and/or hyperalgesia, which are signs of loss of small myelinated (Aδ) fibers or unmyelinated C fibers. Each year, 10% of patients with prediabetes convert to T2DM (Tabák et al., 2012) with progression of existing, as well as new onset, complications. As neuropathy progresses, there is eventual loss of perception of all sensory modalities in a stocking-glove pattern, with poor wound healing, ulcer formation, and, in 15% of patients, amputation (Callaghan et al., 2012a). It is estimated that one-third of the 80 million Americans with prediabetes have neuropathy (Cortez et al., 2014), while the prevalence in the 30 million Americans with T2DM reaches 60% (Callaghan et al., 2015).

The enormity of the problem led us to complete a Cochrane systematic review of all clinical trials of diabetic patients where neuropathy was an outcome measure after instituting tight glycemic control in patients (Callaghan et al., 2012c). Our goal was to understand if targeting glycemia could help restore nerve function. We found that glycemic control actually has little effect on neuropathy development and progression in T2DM patients (Callaghan et al., 2012c), confirming the recommendation of the American Diabetes Association that early intervention with diet and exercise in patients with prediabetes is the best therapeutic option for the prevention of neuropathy (www.ada.org). Our own clinical studies support these guidelines and implicate the metabolic syndrome as the underlying root cause of neuropathy, independent of glycemic status. In a cohort of 2382 participants from the Health, Aging and Body Composition (Health ABC) study, we recently reported that metabolic syndrome components are significantly associated with neuropathy independent of glycemic status (Callaghan et al., 2016a). This recent study also confirms our earlier data from the Lifestyle Intervention Study in prediabetic patients (Smith et al., 2006), where baseline neuropathy most strongly associated with having two or more components of the metabolic syndrome, and was not dependent on glycemic status (Cortez et al., 2014). In these patients, weight loss and improvements in dyslipidemia most closely correlated with improvement in neuropathy (Smith et al., 2006).

Our laboratory uses murine models that most closely resemble human disease to investigate the pathogenesis of neuropathy and identify new therapeutic interventions. We and others have previously reported that C57BL/6J (B6-*wt*) mice fed a high-fat diet (HFD) from lard (pig fat) develop components of the metabolic syndrome, including obesity, impaired glucose tolerance and dyslipidemia, as well as neuropathy (Vincent et al., 2009; Anderson et al., 2014); however, because the background murine strain affects the development of the metabolic syndrome in response to a HFD (Sims et al., 2013; Anderson et al., 2014), it was unknown if HFD-fed B6-*wt* animals provided the most robust and reproducible murine model to simulate human disease. The goals of the current study were two-fold. First, we aimed to identify the optimal murine model of HFD-induced metabolic syndrome and neuropathy across six background/genotype combinations commonly used in experimental diabetes research: BKS-*wt*, BKS-*Lepr^db/+^*, B6-*wt*, B6-*Lepr^db/+^*, BTBR-*wt*, and BTBR-*Lep^ob/+^* (see Materials and Methods for complete description of background strains, *Lepr^db/+^* and *Lep^ob/+^*). Our criteria for the metabolic syndrome were obesity, impaired glucose tolerance and dyslipidemia. Our criteria for murine neuropathy were early and maintained sural and sciatic nerve conduction velocity (NCV) deficits and cutaneous innervation, the same criteria used in prediabetic and T2DM patients to diagnose neuropathy (Smith and Singleton, 2013, 2012). Second, once the optimal murine model of neuropathy and the metabolic syndrome was established, we investigated whether murine HFD-induced nerve dysfunction could be reversed with a dietary reversal paradigm (Sims-Robinson et al., 2016), as we observed in our clinical studies (Smith et al., 2006).

We report that B6-*wt* mice fed a 54% HFD from lard developed the most robust metabolic syndrome components and neuropathy phenotypes across the six murine strains evaluated. Moreover, after 16 weeks on a HFD, mice switched to a standard diet for only 4 weeks exhibited weight loss, improved glucose tolerance, 50% restoration of low density lipoprotein (LDL) and oxidized LDL (oxLDL) cholesterol levels, and complete normalization of NCVs compared with HFD control mice. This model thus provides a basis for future mechanistic studies that will investigate which components of the metabolic syndrome are the primary drivers of neuropathy and that aim to understand how weight loss can improve nerve function.

## RESULTS

### Metabolic phenotyping

**Fig. 1.**
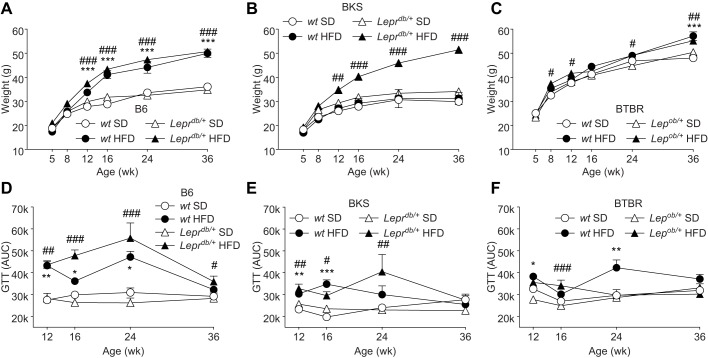
**Longitudinal body weight and glucose tolerance tests.** Body weights were measured for all mice at 5, 8, 12, 16, 24 and 36 weeks (A-C) (*n*=12/group). Glucose tolerance testing (GTT) was performed at 12, 16, 24 and 36 weeks in a cohort of mice from each group (*n*=6/group) (D-F). After a baseline fasting blood glucose measurement, 1 g glucose/kg body weight was injected i.p. and blood glucose was measured at 15, 30, 60 and 120 min post-injection. For statistical analyses, area under the curve (AUC) was calculated for each mouse, and the mean for each time point is displayed. Data are mean±s.e.m. White circles: wild-type mice fed standard diet (SD); white triangles: *Lepr^db/+^* or *Lep^ob/+^* mice fed SD; black circles: wild-type mice fed high-fat diet (HFD); black triangles: *Lepr^db/+^* or *Lep^ob/+^* mice fed HFD. Ordinary two-way ANOVA with Tukey's multiple comparisons test were performed on the four datasets within the individual graphs. *P<0.05, **P<0.01, ***P<0.001, wild-type mice fed HFD versus SD; ^#^*P*<0.05, ^##^*P*<0.01, ^###^*P*<0.001, *Lepr^db/+^* or *Lep^ob/+^* mice fed HFD versus SD.

To investigate and compare the effects of HFD on the metabolic phenotype between each strain, longitudinal changes in body mass, fasting glucose and glucose tolerance were measured. Between 12 and 36 weeks, HFD-fed B6-*wt* and B6-*Lepr^db/+^* mice were significantly heavier than mice fed a standard diet (Fig. 1A); however, on the BKS background, the *Lepr^db/+^* mutation was required for HFD-induced obesity (Fig. 1B). HFD-fed BTBR mice exhibited similar weight gain to their B6 counterparts and did so at equal rates, independent of diet (Fig. 1C). Area under the curve analyses (Fig. 1D-F) of longitudinal glucose tolerance testing (Fig. S1) revealed that HFD-fed mice exhibited varying degrees of impaired glucose tolerance between 12 and 24 weeks, although all responses improved by 36 weeks. Compared with the other strains, HFD-fed B6-*wt* and B6-*Lepr^db/+^* mice displayed a more profound impaired glucose tolerance between 12 and 24 weeks. As predicted, HFD-fed mice, with the exception of B6-*Lepr^db/+^* mice at 16 weeks, did not display severe hyperglycemia when baseline glucose levels were measured (0 min) (Fig. S1).

**Fig. 2.**
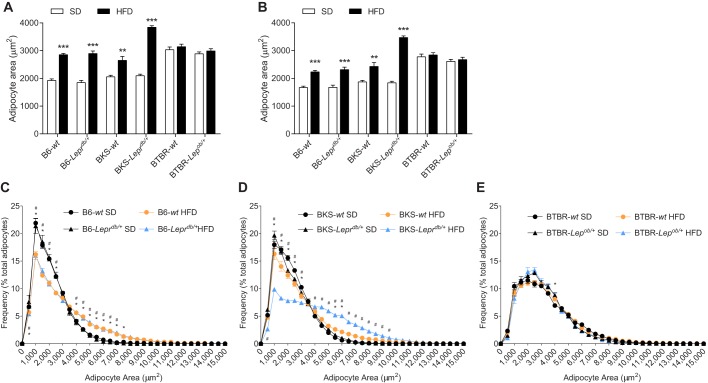
**Adipocyte histomorphometry of epididymal white adipose tissue.** (A,B) At study conclusion, area analysis was performed on adipocytes within epididymal white adipose tissue using MetaMorph software. Graph in A shows mean values whereas that in B shows median values. (C-E) Size–frequency distribution was calculated to account for changes in both adipocyte number and size. Six sections, more than 100 µm apart were averaged per mouse. Data are mean±s.e.m. (median±s.e.m. in B) from *n*=6 mice/group. In A,B, **P<0.01, ***P<0.001, wild-type mice fed HFD versus SD; in C-E, *P≤0.05, wild-type mice fed HFD versus SD; ^#^*P*≤0.05, *Lepr^db/+^* or *Lep^ob/+^* mice fed HFD versus SD.

Upon study conclusion, terminal glycated hemoglobin, fasting blood glucose, plasma insulin, triglycerides and cholesterol were measured (Table 1), and white adipose tissue phenotype was assessed (Fig. 2). Terminal fasting blood glucose and glycated hemoglobin were unaffected by HFD, confirming that mice did not develop a T2DM-like phenotype. HFD-fed B6 and BTBR mice (-*wt*, -*Lepr^db/+^* and -*wt*, *-Lep^ob/+^*, respectively) developed significant hyperinsulinemia, unlike the BKS strain. At 36 weeks, only B6-*wt* HFD mice displayed a robust profile of dyslipidemia, with a 2-fold increase in plasma cholesterol (Table 1). This hypercholesterolemia was reflected in both absolute LDL- and oxLDL-cholesterol (Fig. S2A,D). Fast protein liquid chromatography (FPLC) analyses confirmed no effect of the HFD on very low density lipoprotein (VLDL), LDL or high density lipoprotein (HDL) triglyceride profiles (Fig. S3). Histomorphological analysis of epididymal white adipose tissue revealed that the size–frequency distribution profiles of adipocytes of each strain closely mirrored the pattern observed in weight gain (Fig. 2).

**Table 1.**
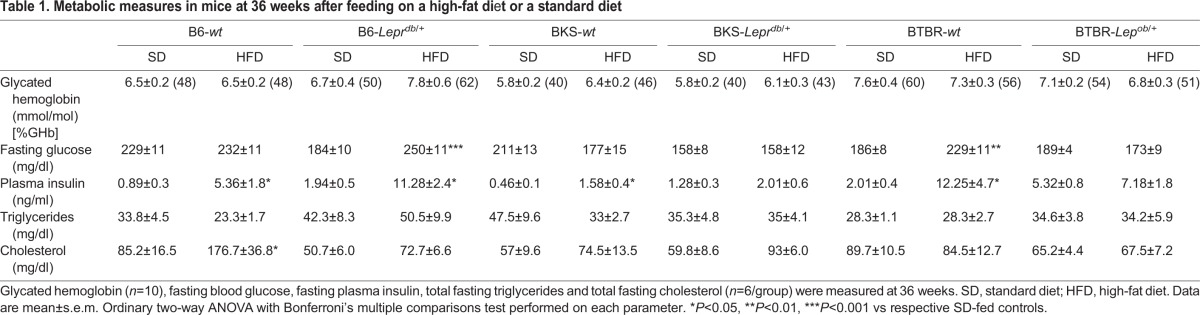
**Metabolic measures in mice at 36 weeks after feeding on a high-fat di**e**t or a standard diet**

### Longitudinal and terminal neuropathy measures

**Fig. 3.**
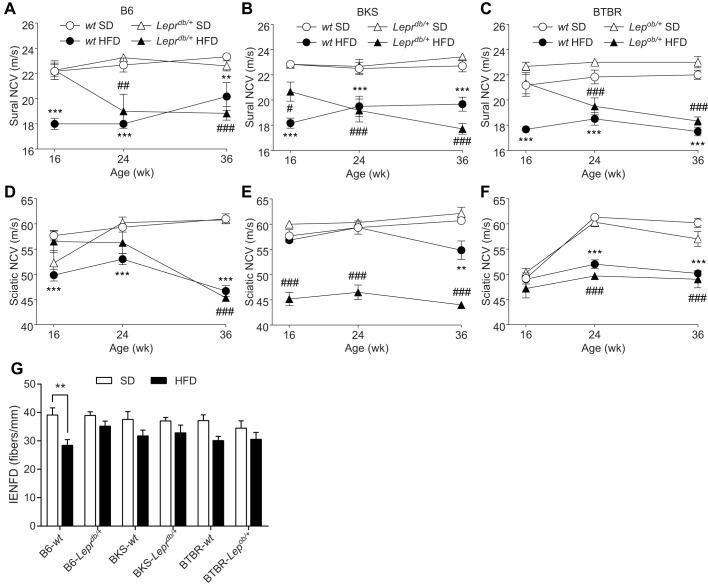
**Measures of nerve fiber function and intra-epidermal nerve fiber density.** Sural (A-C) and sciatic (D-F) nerve conduction velocities (NCVs) were measured at 16, 24 and 36 weeks for all groups (*n*=6/group). Anatomical measures of intra-epidermal nerve fiber density (IENFD) were measured for all groups at 36 weeks of age (G; *n*=8/group). Data are mean±s.e.m. White circles: wild-type mice fed standard diet (SD); white triangles: *db/+* or *ob/+* mice fed SD; black circles: wild-type mice fed HFD; black triangles: *Lepr^db/+^* or *Lep^ob/+^* mice fed HFD. Ordinary two-way ANOVA with Tukey's multiple comparisons test performed on the four datasets within the individual figures (A-F). Ordinary two-way ANOVA with Bonferroni's multiple comparisons test were performed on IENFD (G). **P<0.01, ***P<0.001, wild-type mice fed HFD versus SD; ^#^*P*<0.05, ^##^*P*<0.01, ^###^*P*<0.001, *Lepr^db/+^* or *Lep^ob/+^* mice fed HFD versus SD.

To investigate longitudinal strain-dependent effects of the HFD on peripheral nerve function, sural and sciatic NCVs, measures of sensory and motor large fiber function, respectively, were measured at 16, 24 and 36 weeks (Fig. 3). While all HFD-fed mice displayed deficits in sural and sciatic NCV by 36 weeks, notable strain-dependent differences were evident earlier. When comparing longitudinal sural NCV measures, with the exception of B6-*Lepr^db/+^* and BTBR*-Lep^ob/+^* mice at 16 weeks, all HFD-fed mice exhibited decreased sural NCV (Fig. 3A-C). Sciatic NCV measures also showed clear differences between strains. Unlike HFD-fed B6-*Lepr^db/+^* mice, HFD-fed B6-*wt* mice displayed a significant decrease in sciatic NCV at 16-24 weeks (Fig. 3D). Compared with BKS-*wt* mice, HFD-fed BKS-*Lepr^db/+^* mice showed profound sciatic NCV deficits at 16 weeks (Fig. 3E). Finally, HFD-fed BTBR-*wt* and -*Lep^ob/+^* mice showed similar deficits at 24-36 weeks, but when measured earlier at 16 weeks, no significant difference was observed relative to mice fed the standard diet (Fig. 3F). Overall, only HFD-fed BKS-*Lepr^db/+^* and B6-*wt* mice developed and maintained early sural and sciatic NCV deficits.

Having established clear differences in HFD-induced large fiber dysfunction between strains, we sought to identify changes in distal small fiber innervation of the skin. Intra-epidermal nerve fiber density was significantly decreased in HFD B6-*wt* mice compared with B6-*wt* controls, while such changes were not significantly affected by a HFD in any of the other groups (Fig. 3G). Moving proximally, sural nerve myelinated fiber density, a measure of sensory fiber architecture within the sural nerve, was not significantly affected by HFD in any of the groups (Fig. S4).

### Effects of dietary reversal

**Fig. 4.**
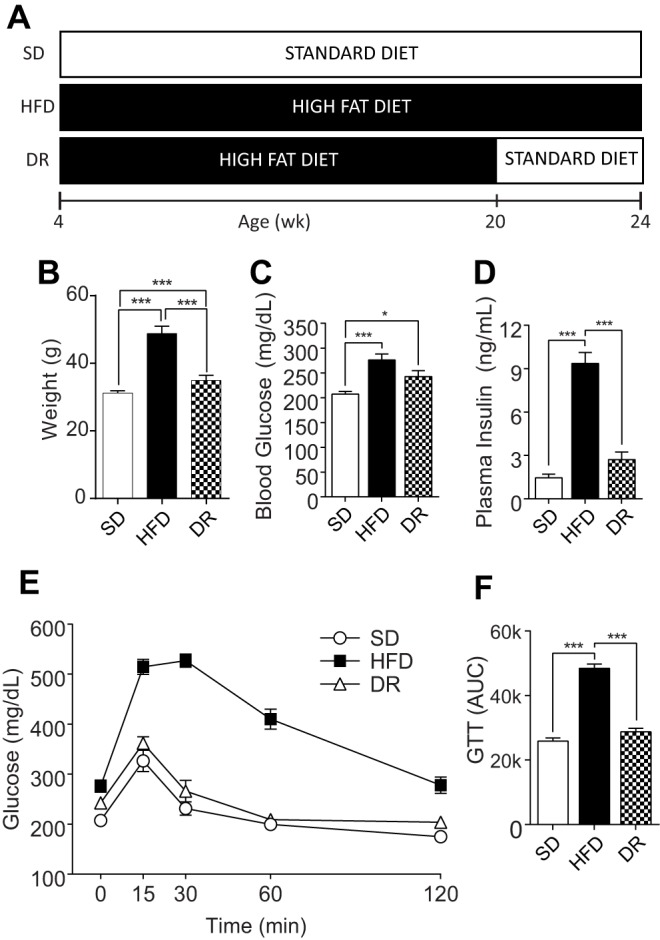
**Dietary reversal following 16 weeks of high-fat feeding lowers body weight and restores insulin sensitivity.** (A) Dietary reversal study design. C57BL/6J mice were fed a 10% standard diet (SD) or a 54% HFD until 24 weeks of age. A cohort of HFD mice were HFD fed until 20 weeks and then placed on standard diet for the remaining 4 weeks (DR). Terminal body weight (B; *n*=8), fasting blood glucose (C; *n*=8) and plasma insulin (D; *n*=6) were measured, and glucose tolerance testing (GTT) was performed (E; *n*=8). For statistical analyses, area under the blood glucose curve (AUC) was calculated for each mouse, and the mean for each time point calculated (F). Data are mean±s.e.m. Ordinary one-way ANOVA with Tukey's multiple comparisons test were performed on the three datasets in B,C,D,F. *P<0.05, ***P<0.001.

**Fig. 5.**
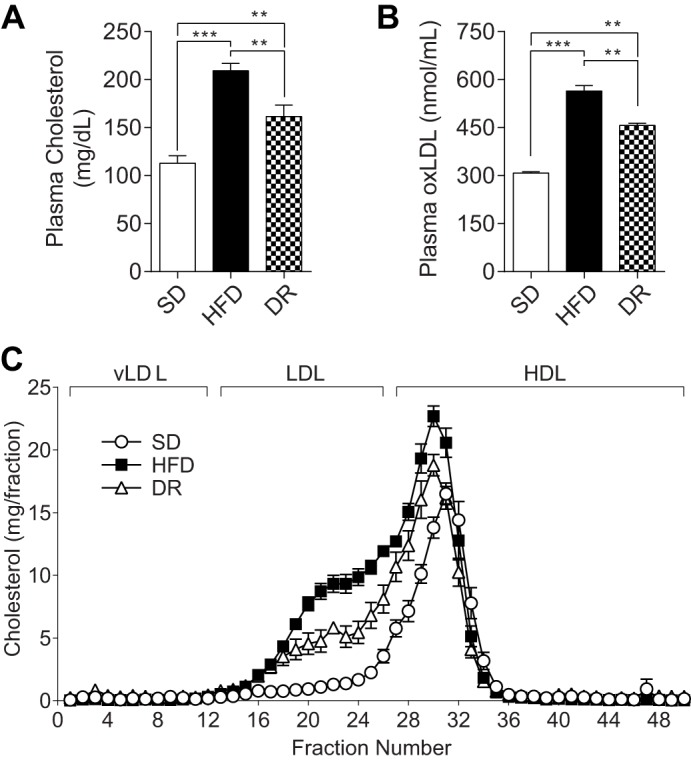
**Dietary reversal following 16 weeks of HFD lowers plasma cholesterol and oxidized LDL.** Total fasting cholesterol (A), oxidized low density lipoprotein (oxLDL) (B) and lipoprotein profiles (C) were assessed at 24 weeks. For lipoprotein profiles, plasma was fractionated by fast protein liquid chromatography and total cholesterol in each fraction was measured. Fractions 1-12 represent very low density lipoproteins (vLDL); 13-26, LDL; 27-50, high density lipoproteins (HDL). White circles: standard diet (SD); black squares: high-fat diet (HFD); white triangles: diet reversal (DR), comprising HFD for 4-20 weeks, switched to SD for 20-24 weeks. In all panels, *n*=6. Data are mean±s.e.m. Ordinary one-way ANOVA with Tukey's multiple comparisons test were performed on the three datasets in A and B. **P<0.01, ***P<0.001.

As HFD-fed B6-*wt* mice develop obesity, impaired glucose tolerance and NCV deficits by 16 weeks, and progress to exhibit intra-epidermal nerve fiber loss, this mouse strain/genotype serves as an optimal murine model of HFD-induced metabolic syndrome and neuropathy. Using a separate cohort of mice, we next investigated if murine HFD-induced nerve dysfunction could be reversed with a dietary reversal paradigm (Fig. 4A) (Sims-Robinson et al., 2016), similar to results obtained in our clinical studies (Smith et al., 2006). B6-*wt* mice on the dietary reversal feeding strategy received HFD from 4-20 weeks of age followed by control chow from 20-24 weeks of age (Fig. 4A). As predicted, by 24 weeks, HFD-fed mice exhibited weight gain, elevated fasting blood glucose, hyperinsulinemia, and impaired glucose tolerance compared with standard diet-fed controls, while dietary reversal mice exhibited significant weight loss, with improvements in fasting blood glucose and insulin levels, and impaired glucose tolerance when compared with age-matched HFD-fed controls (Fig. 4B-F). Lipid profiling revealed significant HFD-induced increases in total cholesterol and LDL cholesterol at 16 and 24 weeks, and in oxLDL at 24 weeks (Fig. 5, Fig. S5). The HFD-induced increases in these cholesterol parameters were corrected by ∼50% in dietary reversal mice at 24 weeks (Fig. 5). In contrast, total triglycerides were elevated in HFD mice at 16 weeks, but this did not persist to 24 weeks (Fig. S6). Together, these metabolic data indicate that dietary reversal mice lost weight, had improved insulin sensitivity, and exhibited a 50% reversal of the HFD-induced increases in plasma cholesterol and oxLDL.

**Fig. 6.**
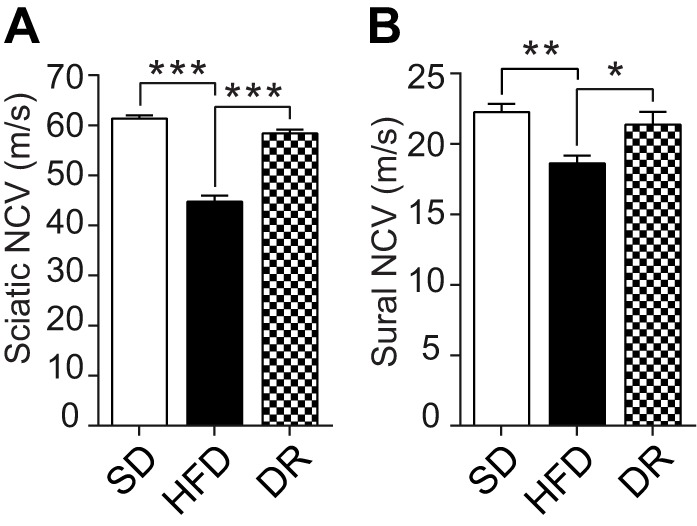
**Dietary reversal following 16 weeks of high-fat feeding restores nerve function.** Sciatic (A) and sural (B) nerve conduction velocities (NCVs) were measured at 24 weeks. Data are mean±s.e.m. (*n*=8/group). Ordinary one-way ANOVA with Tukey's multiple comparisons test were performed on the three datasets in A and B. *P<0.05, **P<0.01, ***P<0.001.

To investigate the effects of these metabolic changes on neuropathy, terminal NCVs were assessed. Both sciatic and sural NCVs were decreased 30% and 16% in HFD-fed mice relative to standard diet-fed mice, respectively (Fig. 6A,B); however, dietary reversal mice exhibited significantly improved NCVs compared with HFD-fed mice, with restoration of 82% and 76% of the sciatic and sural NCV deficits, respectively. These data indicate that HFD-induced sensory and motor large fiber dysfunction can be reversed via a short-term dietary reversal paradigm.

## DISCUSSION

Mouse strain-dependent variability in neuropathy phenotypes has been documented in T2DM (O'Brien et al., 2014b); however, similar information regarding HFD-induced neuropathy in a murine model of prediabetes and metabolic syndrome is limited (Anderson et al., 2014). We present the first longitudinal, intra-experimental comparison of HFD-induced peripheral neuropathy across multiple mouse strains. All six strain/genotype combinations examined developed impaired glucose tolerance and large-fiber neuropathy on a HFD; however, these changes developed earlier and more consistently in B6-*wt* mice. B6-*wt* mice displayed obesity, hyperinsulinemia, dyslipidemia (hypercholesterolemia) and elevated oxLDL, with concomitant sensory and motor large-fiber dysfunction and distal small-fiber degeneration. In contrast, while HFD-fed B6-*Lepr^db/+^* mice were obese, these animals did not display elevated LDL or oxLDL cholesterol, and the mutation did not confer a greater neuropathy phenotype. The BKS strain required genetic manipulation of leptin receptor signaling (-*Lepr^db/+^*) for weight gain and early neuropathy development, while all mice on the BTBR background gained weight at equal rates, independent of diet, making these less-desirable models of prediabetes, metabolic syndrome and neuropathy. Using the B6-*wt* HFD model, our investigation of whether HFD-induced nerve dysfunction could be reversed with a simple dietary reversal paradigm (Sims-Robinson et al., 2016) revealed that after 16 weeks on a HFD, mice switched to a standard diet for only 4 weeks exhibited complete normalization of large-fiber function (sural and sciatic NCVs) accompanied by weight loss, improved glucose tolerance (as an indicator of improved insulin sensitivity), and 50% restoration of total, LDL and oxLDL cholesterol compared with levels in HFD control mice.

Several interesting observations were evident regarding the effects of HFD-induced obesity between mouse strains. First, the resistance of BKS-*wt* mice to HFD-induced obesity agrees with a previous report that BKS mice restrict food intake and increase activity in response to a HFD (Sims et al., 2013). Second, our data indicate that a *Lepr^db/+^* or *Lep^ob/+^* mutation is not additive to weight gain in B6 or BTBR mice, but in BKS mice, the *Lepr^db/+^* mutation is necessary for HFD-induced obesity. Finally, in BTBR mice, the HFD did not increase body weight compared with mice on the standard diet. It remains unclear whether a behavioral modification underlies the failure of BTBR mice to develop HFD-induced obesity, as seen in BKS-*wt* mice. White adipose tissue size–frequency distribution profiles mirrored these body weight changes, collectively reinforcing the link between obesity, white adipose tissue hypertrophy and metabolic abnormalities (Lumeng and Saltiel, 2011; Gregor and Hotamisligil, 2011). Unlike the other strains examined, B6-*wt* mice developed early obesity, dyslipidemia and impaired glucose tolerance, as anticipated (Sims et al., 2013; Li et al., 2010; Mielke et al., 2006; Muller et al., 2013), identifying this murine model as the one that most closely replicates human disease. The apparent improvements in insulin sensitivity at 36 weeks (in all HFD-fed strains) were somewhat unexpected; however, a prolonged HFD has been reported to result in improved impaired glucose tolerance (Muller et al., 2013). Moreover, plasma insulin levels remained high in our animals, suggesting that our use of a lower dose of glucose for glucose tolerance testing may have compounded any potential HFD duration-related effects [1 g/kg body weight as per Diabetic Complications Consortium protocol, compared with 2 g/kg body weight in other studies (Sims et al., 2013; Mielke et al., 2006)].

Although strain differences in HFD-induced metabolic syndrome components have been previously reported (Sims et al., 2013; Anderson et al., 2014), information regarding strain differences in HFD-induced neuropathy has been limited (Anderson et al., 2014). Most HFD-induced neuropathy studies have used B6 mice, with cross-study comparisons complicated by age at initiation of high-fat feeding, fat content ranging between 40 and 60%, and different dietary sources of fat (lard, coconut oil, vegetable shortening etc.) (see O'Brien et al., 2014b for discussion). In this vein, our B6 data are in agreement with our previous data (Vincent et al., 2009) and with the recent report (Yorek et al., 2015) that male B6 mice fed a 45-60% HFD from lard show reductions in NCV and intra-epidermal nerve fiber density within as few as 12 weeks of HFD feeding. In contrast, data from Groover et al. (2013) demonstrate that male B6 mice fed a 54% HFD from hydrogenated vegetable shortening for 12 weeks do not develop these large- or small-fiber deficits. These data suggest that animal fat is required for HFD-induced large- and small-fiber changes in male B6 mice. Interestingly, Obrosova et al. (2007) reported that female B6 mice fed a 58% HFD from hydrogenated coconut oil for 16 weeks develop NCV deficits, but intra-epidermal nerve fiber density is not affected. The roles of fat source and gender in this discrepancy, however, remain to be determined.

When comparing neuropathy progression between strains, we observed that in HFD-fed mice, similar to weight gain, the *Lepr^db/+^* mutation was required for sciatic NCV reductions. These data, along with the fact that BKS mice homozygous for the leptin receptor mutation (*Lepr^db/db^*) exhibit sural and sciatic NCV deficits by 16 weeks (Ii et al., 2005; Wang et al., 2011; Kan et al., 2012; Hur et al., 2015), may suggest that genetic disruption of leptin signaling is required for large-fiber dysfunction, independent of obesity. Interestingly, unlike the other strains, sciatic NCV measures in BTBR mice fed a standard diet at 16 weeks were lower when compared with the other strains. Relative to sural NCVs, sciatic NCV measures the function of more-heavily myelinated motor nerve fibers, and we previously reported an age-related increase in sciatic NCV from 9–13 weeks in control chow-fed BTBR-*Lep^ob/ob^* mice (O'Brien et al., 2014a), suggestive of maturation-related myelination. Thus, it is possible that a similar maturation effect contributed to the slow sciatic NCVs in control chow-fed BTBR mice. Further studies, including g-ratio calculations in nerves, are required to assess potential HFD-induced hypomyelination and its contribution to the observed NCV deficits. Changes in NCVs often reflect underlying structural pathology, including atrophy, demyelination and loss of fiber density (Boulton et al., 2004). We report loss of small fibers in distal skin with a HFD, with no significant change in nerve fiber density moving proximally along the sural nerve (sural nerve myelinated fiber density). This is consistent with clinical evidence that changes in small nerve fibers are among the earliest detectable sign of neuropathy, and that neuropathy associated with impaired glucose tolerance is milder in its early stages when compared with that associated with T2DM (Kluding et al., 2012).

In prediabetic patients with metabolic syndrome, both dietary modification and exercise improve several components of the metabolic syndrome and neuropathic features (Smith et al., 2006; Singleton et al., 2015a,b). Moreover, because exercise alone significantly improves neuropathic symptoms and increases cutaneous nerve fiber branching in T2DM patients (Kluding et al., 2012), we next assessed whether dietary modification could similarly restore nerve function in HFD-fed B6-*wt* mice, which develop a greater number of characteristics of the metabolic syndrome and peripheral neuropathy than the other strains. A similar study investigated the role of dietary reversal in HFD-fed female B6 mice (Obrosova et al., 2007); however, since female mice are resistant to HFD-induced obesity (Pettersson et al., 2012; Louet et al., 2004), the current study focused on determining the effect of dietary reversal on neuropathy in male B6 mice. NCVs were chosen as end points based on EASD Study Group recommendations (Biessels et al., 2014) and the Toronto Consensus Criteria (Tesfaye et al., 2010). Following dietary reversal, we observed almost complete normalization of not only weight, lipid levels and insulin resistance, but also sural and sciatic NCV measures. It is important to note that HFD feeding began before peripheral nervous system maturation. We are conducting a comparative study to assess whether the dietary reversal paradigm has similar salutatory effects when HFD is given after nervous system maturation.

In the current study, triglycerides were not elevated by sustained HFD feeding, nor were they affected by dietary reversal. These data suggest that triglycerides may be less important in mediating HFD-induced peripheral neuropathy than in mediating T2DM neuropathy (Wiggin et al., 2009). What did associate with HFD-induced large nerve fiber dysfunction was a different form of dyslipidemia, characterized by hypercholesterolemia and elevated oxLDL. We recently reported that while pioglitazone treatment normalized glycemia and triglyceride levels in BKS mice homozygous for the *Lepr^db/+^* mutation, there was no decrease in body weight or improvement in total and LDL cholesterol levels, or sural and sciatic NCVs in this model of T2DM (Hur et al., 2015). In a separate study, we also demonstrated direct oxLDL-mediated dorsal root ganglia neuronal injury (Vincent et al., 2009). Thus, our current and previously reported murine data collectively support our findings in humans (Callaghan et al., 2012c, 2016a; Smith et al., 2006; Singleton et al., 2015a,b) and point towards a role for components of the metabolic syndrome in HFD-induced large fiber dysfunction. Our future studies will determine if a constellation of metabolic improvements is required for improved nerve function, or if one single parameter has a greater beneficial effect than the others.

In summary, this is the first comparative study of the effect of strain on HFD-induced neuropathy in animals with one or more components of the metabolic syndrome. We conclude that B6-*wt* mice are the most robust model of HFD-induced neuropathy, with essential components of the metabolic syndrome, specifically obesity, prediabetes and dyslipidemia. Furthermore, we report that HFD-induced dysfunction of sensory and motor large fibers can be reversed via a short-term dietary reversal paradigm. We contend that visceral adiposity, hypercholesterolemia and prediabetes combine to injure the peripheral nervous system in HFD-fed murine models. These findings suggest that new guidelines for strict and specific dietary control could provide the first disease-modifying treatment for early neuropathy. In addition, this model provides the basis for the mechanistic studies required to investigate the role of the components of the metabolic syndrome in mediating peripheral nerve damage and for the development of future pharmacological adjuncts to existing therapies for prediabetic and diabetic neuropathy.

## MATERIALS AND METHODS

### Mice/diet

Male mice consisted of six strains/genotypes: BKS-*wt*, BKS-*Lepr^db/+^*, B6-*wt*, B6-*Lepr^db/+^*, BTBR-*wt*, and BTBR*-Lep^ob/+^* mice (C57BLKS/J #000662, BKS.Cg-Dock7 m +/+ Leprdb/J #000642, C57BL/6J #000664, B6.BKS(D)-Leprdb/J #000697, BTBR T+ Itpr3tf/J #002282 and BTBR.Cg-Lepob/WiscJ #004824, respectively; Jackson Laboratory, Bar Harbor, ME, USA). Homozygous mutations in leptin (*Lep^ob/ob^*) or the leptin receptor (*Lepr^db/db^*) result in compromised leptin signaling, chronic hyperphagia and the development of T2DM. These mice are therefore widely used as genetic models of T2DM. Mice with heterozygous mutations (*Lep^ob/+^/Lepr^db/+^*) do not develop diabetes, and are widely used as non-diabetic controls in these studies (see O'Brien et al., 2014b for comprehensive review). Notably, BKS mice are a substrain derived from B6, sharing ∼70% of the B6 genome. Mice were fed a standard diet or a HFD (#D12540-B, 10% kcal fat or #05090701, 54% kcal fat from lard, respectively; Research Diets, New Brunswick, NJ, USA). Mice were randomly assigned to a treatment group upon arrival and placed on standard diet or HFD from 4 weeks of age. The strain comparison study was conducted once, *n*=12/group. The dietary reversal study was conducted once, *n*=8/group (Fig. 4A), shortly after completion of the strain comparison study.

Animals were maintained in a pathogen-free environment and cared for by the University of Michigan Unit for Laboratory Animal Medicine. All protocols were approved by the University of Michigan University Committee on Use and Care of Animals and followed Diabetic Complications Consortium guidelines (https://www.diacomp.org/shared/protocols.aspx).

### Metabolic/lipid phenotyping

Body weights, fasting blood glucose (4 h fast), and glucose tolerance were assessed longitudinally; fasting blood glucose was measured using an AlphaTrak Glucometer (Abbott Laboratories, Abbott Park, IL, USA) and blood glucose was monitored over 2 h post-administration of a glucose bolus (1 g/kg body weight i.p.). Terminal glycated hemoglobin was measured by the Chemistry Core at the Michigan Diabetes Research Center. A Mouse Metabolic Phenotyping Center (MMPC; Vanderbilt University, Nashville, TN; University of Cincinnati, Cincinnati, OH, USA) determined fasting plasma insulin, triglycerides, and cholesterol and performed fast protein liquid chromatography (FPLC) analysis for cholesterol and triglyceride lipoprotein profiles. Plasma was diluted 1:200 and oxLDL was quantified via ELISA as per the manufacturer's instructions (#CSB-E07933 m, American Research Products, Waltham, MA, USA). Histomorphological assessment of epididymal white adipose tissue was determined in a blinded manner using MetaMorph Image Analysis Software v.7.7.0.8 (Molecular Devices, Sunnyvale, CA, USA) as previously described (Parlee et al., 2014).

### Neuropathy phenotyping

Neuropathy phenotyping was performed according to Diabetic Complications Consortium guidelines (Biessels et al., 2002; Biessels and Kappelle, 2005). Nerve conductions were conducted according to Oh et al. (2010). For sural nerve NCV, recording electrodes were placed on the dorsum of the foot and stimulating electrodes on the ankle. Onset latency (ms) of the sensory nerve action potential after supramaximal antidromic stimulation of the sural nerve at the ankle was divided into the distance between the recording and stimulating electrodes (mm) to calculate the sural NCV (m/s). For sciatic-tibial motor NCV, recording electrodes were placed on the dorsum of the foot and the nerve was orthodromically stimulated first at the ankle, then at the sciatic notch. The distance between the two sites of stimulation (mm) was divided by the difference between the two onset latencies of the compound muscle action potentials (ms) to calculate the sciatic-tibial NCV (m/s). Intra-epidermal nerve fiber density was determined according to Cheng et al. (2012). Briefly, footpads from the plantar surface of the hind paw were dissected, fixed for 3 h in Zamboni's fixative (2% paraformaldehyde, 0.2% picric acid in 0.1 M phosphate buffer), then washed with 30% sucrose in 0.1 M phosphate buffer saline. Footpads were cryo-embedded in OCT mounting medium and sectioned at 30 μm for immunohistochemistry. Sections were labeled with PGP9.5 (1:1000, cat. no. 7863-0504, ABD Serotec, Raleigh, NC, USA) pan-axonal antibody and goat anti-rabbit 488 secondary antibody (1:2000, cat. no. A-11034, Thermo Fisher Scientific, Waltham, MA, USA) to identify cutaneous nerve fibers. Sections were imaged using a Leica SP5 confocal microscope (20×1.2 water-immersion objective, 1024×1024 pixels resolution). Three *z*-series were captured per section, each consisting of ≥20 images, with 2.0 μm optical thickness. Each *z*-series was flattened using the MetaMorph (v.7.7.0.8, Molecular Devices) maximum stack arithmetic feature and the number of fibers per linear millimeter of epidermis was determined. For sural nerve myelinated fiber density, samples were immersed in 4% paraformaldehyde, 2.5% glutaraldehyde in PBS, pH 7.4, rinsed and embedded in resin. Three 0.5-µm-thick sections were cut from the distal and proximal end of the sural nerve and stained with Toluidine Blue. Images were captured using a Nikon Microphot-FXA upright microscope and analyzed in a blinded manner using MetaMorph software. Samples were imaged with a 60× oil objective. The number of myelinated fibers and the area of each nerve section were determined manually. The ratio of fibers per area was averaged for six sections to determine final sural nerve myelinated fiber density.

### Statistical analyses

Based on previously published (Vincent et al., 2009; Hur et al., 2015; Hinder et al., 2017) studies, our reported sample sizes of *n*=12/group for the strain comparison study and *n*=8/group for the dietary reversal study were adequately powered to detect significant differences in the respective parameters. Statistical analyses were performed using GraphPad Prism v.6 (GraphPad Software, La Jolla, CA, USA). Longitudinal comparisons between multiple groups were performed using two-way ANOVA with Tukey's post-test for multiple comparisons. Cross-section comparisons between multiple groups were performed using one-way ANOVA with Tukey's or Bonferroni's post-test for multiple comparisons, as applicable (Festing and Altman, 2002). Assumptions about Gaussian distribution of data were made as previously described (Russell et al., 1999). Accordingly, non-parametric tests were not required for any data analyses. Significance was assigned when *P*<0.05. Values represent the mean±s.e.m.

## References

[DMM028530C1] Anderson N. J., King M. R., Delbruck L. and Jolivalt C. G. (2014). Role of insulin signaling impairment, adiponectin and dyslipidemia in peripheral and central neuropathy in mice. *Dis. Model. Mech.* 7, 625-633. 10.1242/dmm.015750 24764191 PMC4036470

[DMM028530C2] Biessels G. J. and Kappelle L. J. (2005). Increased risk of Alzheimer's disease in Type II diabetes: insulin resistance of the brain or insulin-induced amyloid pathology? *Biochem. Soc. Trans.* 33, 1041-1044. 10.1042/BST0331041 16246041

[DMM028530C3] Biessels G. J., Van Der Heide L. P., Kamal A., Bleys R. L. A. W. and Gispen W. H. (2002). Ageing and diabetes: implications for brain function. *Eur. J. Pharmacol.* 441, 1-14. 10.1016/S0014-2999(02)01486-3 12007915

[DMM028530C4] Biessels G. J., Bril V., Calcutt N. A., Cameron N. E., Cotter M. A., Dobrowsky R., Feldman E. L., Fernyhough P., Jakobsen J., Malik R. A. et al. (2014). Phenotyping animal models of diabetic neuropathy: a consensus statement of the diabetic neuropathy study group of the EASD (Neurodiab). *J. Peripher. Nerv. Syst.* 19, 77-87. 10.1111/jns5.12072 24934510 PMC4303044

[DMM028530C5] Boulton A. J. M., Malik R. A., Arezzo J. C. and Sosenko J. M. (2004). Diabetic somatic neuropathies. *Diabetes Care* 27, 1458-1486. 10.2337/diacare.27.6.1458 15161806

[DMM028530C6] Callaghan B. C., Cheng H. T., Stables C. L., Smith A. L. and Feldman E. L. (2012a). Diabetic neuropathy: clinical manifestations and current treatments. *Lancet Neurol.* 11, 521-534. 10.1016/S1474-4422(12)70065-0 22608666 PMC4254767

[DMM028530C7] Callaghan B. C., Hur J. and Feldman E. L. (2012b). Diabetic neuropathy: one disease or two? *Curr. Opin. Neurol.* 25, 536-541. 10.1097/WCO.0b013e328357a797 22892951 PMC4239661

[DMM028530C8] Callaghan B. C., Little A. A., Feldman E. L. and Hughes R. A. (2012c). Enhanced glucose control for preventing and treating diabetic neuropathy. *Cochrane Database Syst. Rev.* 6, CD007543 10.1002/14651858.cd007543.pub2 PMC4048127 22696371

[DMM028530C9] Callaghan B. C., Price R. S. and Feldman E. L. (2015). Distal symmetric polyneuropathy: a review. *JAMA* 314, 2172-2181. 10.1001/jama.2015.13611 26599185 PMC5125083

[DMM028530C10] Callaghan B. C., Xia R., Banerjee M., De Rekeneire N., Harris T. B., Newman A. B., Satterfield S., Schwartz A. V., Vinik A. I., Feldman E. L. et al. (2016a). Metabolic syndrome components are associated with symptomatic polyneuropathy independent of glycemic status. *Diabetes Care* 39, 801-807. 10.2337/dc16-0081 26965720 PMC4839175

[DMM028530C11] Callaghan B. C., Xia R., Reynolds E., Banerjee M., Rothberg A. E., Burant C. F., Villegas-Umana E., Pop-Busui R. and Feldman E. L. (2016b). Association between metabolic syndrome components and polyneuropathy in an obese population. *JAMA Neurol.* 73, 1468-1476. 10.1001/jamaneurol.2016.3745 27802497 PMC5217829

[DMM028530C12] Cheng H. T., Dauch J. R., Hayes J. M., Yanik B. M. and Feldman E. L. (2012). Nerve growth factor/p38 signaling increases intraepidermal nerve fiber densities in painful neuropathy of type 2 diabetes. *Neurobiol. Dis.* 45, 280-287. 10.1016/j.nbd.2011.08.011 21872660 PMC3225563

[DMM028530C13] Cortez M., Singleton J. R. and Smith A. G. (2014). Glucose intolerance, metabolic syndrome, and neuropathy. *Handb. Clin. Neurol.* 126, 109-122. 10.1016/B978-0-444-53480-4.00009-6 25410218

[DMM028530C14] Festing M. F. W. and Altman D. G. (2002). Guidelines for the design and statistical analysis of experiments using laboratory animals. *ILAR J.* 43, 244-258. 10.1093/ilar.43.4.244 12391400

[DMM028530C15] Gregor M. F. and Hotamisligil G. S. (2011). Inflammatory mechanisms in obesity. *Annu. Rev. Immunol.* 29, 415-445. 10.1146/annurev-immunol-031210-101322 21219177

[DMM028530C16] Groover A. L., Ryals J. M., Guilford B. L., Wilson N. M., Christianson J. A. and Wright D. E. (2013). Exercise-mediated improvements in painful neuropathy associated with prediabetes in mice. *Pain* 154, 2658-2667. 10.1016/j.pain.2013.07.052 23932909 PMC3844098

[DMM028530C111] Hinder L., Park M., Rumora A. E., Hur J., Eichinger F., Pennathur S., Kretzler M., Brosius F. C. III and Feldman E. L. (2017). Comparative RNA-Seq transcriptome analyses reveal distinct metabolic pathways in diabetic nerve and kidney disease. *J. Cell. Mol. Med.* doi: 10.1111/jcmm.13136 10.1111/jcmm.13136 PMC5571536 28272773

[DMM028530C17] Hur J., Dauch J. R., Hinder L. M., Hayes J. M., Backus C., Pennathur S., Kretzler M., Brosius F. C. III and Feldman E. L. (2015). The metabolic syndrome and microvascular complications in a murine model of type 2 diabetes. *Diabetes* 64, 3294-3304. 10.2337/db15-0133 25979075 PMC4542440

[DMM028530C18] Ii M., Nishimura H., Kusano K. F., Qin G., Yoon Y. S., Wecker A., Asahara T. and Losordo D. W. (2005). Neuronal nitric oxide synthase mediates statin-induced restoration of vasa nervorum and reversal of diabetic neuropathy. *Circulation* 112, 93-102. 10.1161/CIRCULATIONAHA.104.511964 15983249

[DMM028530C19] Kan M., Guo G. F., Singh B., Singh V. and Zochodne D. W. (2012). Glucagon-like peptide 1, insulin, sensory neurons, and diabetic neuropathy. *J. Neuropathol. Exp. Neurol.* 71, 494-510. 10.1097/NEN.0b013e3182580673 22588388

[DMM028530C20] Kluding P. M., Pasnoor M., Singh R., Jernigan S., Farmer K., Rucker J., Sharma N. K. and Wright D. E. (2012). The effect of exercise on neuropathic symptoms, nerve function, and cutaneous innervation in people with diabetic peripheral neuropathy. *J. Diabetes Complications* 26, 424-429. 10.1016/j.jdiacomp.2012.05.007 22717465 PMC3436981

[DMM028530C21] Li P., Lu M., Nguyen M. T. A., Bae E. J., Chapman J., Feng D., Hawkins M., Pessin J. E., Sears D. D., Nguyen A.-K. et al. (2010). Functional heterogeneity of CD11c-positive adipose tissue macrophages in diet-induced obese mice. *J. Biol. Chem.* 285, 15333-15345. 10.1074/jbc.M110.100263 20308074 PMC2865288

[DMM028530C22] Louet J.-F., Lemay C. and Mauvais-Jarvis F. (2004). Antidiabetic actions of estrogen: insight from human and genetic mouse models. *Curr. Atheroscler Rep.* 6, 180-185. 10.1007/s11883-004-0030-9 15068742

[DMM028530C23] Lumeng C. N. and Saltiel A. R. (2011). Inflammatory links between obesity and metabolic disease. *J. Clin. Invest.* 121, 2111-2117. 10.1172/JCI57132 21633179 PMC3104776

[DMM028530C24] Mielke J. G., Nicolitch K., Avellaneda V., Earlam K., Ahuja T., Mealing G. and Messier C. (2006). Longitudinal study of the effects of a high-fat diet on glucose regulation, hippocampal function, and cerebral insulin sensitivity in C57BL/6 mice. *Behav. Brain Res.* 175, 374-382. 10.1016/j.bbr.2006.09.010 17081630

[DMM028530C25] Muller A. P., Dietrich Mde O., Martimbianco De Assis A., Souza D. O. and Portela L. V. (2013). High saturated fat and low carbohydrate diet decreases lifespan independent of body weight in mice. *Longev Healthspan* 2, 10 10.1186/2046-2395-2-10 24472284 PMC3922950

[DMM028530C26] O'Brien P. D., Hur J., Hayes J. M., Backus C., Sakowski S. A. and Feldman E. L. (2014a). BTBR ob/ob mice as a novel diabetic neuropathy model: neurological characterization and gene expression analyses. *Neurobiol. Dis.* 73C, 348-355. 10.1016/j.nbd.2014.10.015 PMC4416075 25447227

[DMM028530C27] O'Brien P. D., Sakowski S. A. and Feldman E. L. (2014b). Mouse models of diabetic neuropathy. *ILAR J.* 54, 259-272. 10.1093/ilar/ilt052 24615439 PMC3962259

[DMM028530C28] Obrosova I. G., Ilnytska O., Lyzogubov V. V., Pavlov I. A., Mashtalir N., Nadler J. L. and Drel V. R. (2007). High-fat diet induced neuropathy of pre-diabetes and obesity: effects of “healthy” diet and aldose reductase inhibition. *Diabetes* 56, 2598-2608. 10.2337/db06-1176 17626889

[DMM028530C29] Ogden C. L., Carroll M. D., Kit B. K. and Flegal K. M. (2014). Prevalence of childhood and adult obesity in the United States, 2011-2012. *JAMA* 311, 806-814. 10.1001/jama.2014.732 24570244 PMC4770258

[DMM028530C30] Oh S. S., Hayes J. M., Sims-Robinson C., Sullivan K. A. and Feldman E. L. (2010). The effects of anesthesia on measures of nerve conduction velocity in male C57Bl6/J mice. *Neurosci. Lett.* 483, 127-131. 10.1016/j.neulet.2010.07.076 20691755 PMC2941214

[DMM028530C31] Parlee S. D., Lentz S. I., Mori H. and Macdougald O. A. (2014). Quantifying size and number of adipocytes in adipose tissue. *Methods Enzymol.* 537, 93-122. 10.1016/B978-0-12-411619-1.00006-9 24480343 PMC4069255

[DMM028530C32] Pettersson U. S., Waldén T. B., Carlsson P.-O., Jansson L. and Phillipson M. (2012). Female mice are protected against high-fat diet induced metabolic syndrome and increase the regulatory T cell population in adipose tissue. *PLoS ONE* 7, e46057 10.1371/journal.pone.0046057 23049932 PMC3458106

[DMM028530C33] Russell J. W., Sullivan K. A., Windebank A. J., Herrmann D. N. and Feldman E. L. (1999). Neurons undergo apoptosis in animal and cell culture models of diabetes. *Neurobiol. Dis.* 6, 347-363. 10.1006/nbdi.1999.0254 10527803

[DMM028530C34] Sims E. K., Hatanaka M., Morris D. L., Tersey S. A., Kono T., Chaudry Z. Z., Day K. H., Moss D. R., Stull N. D., Mirmira R. G. et al. (2013). Divergent compensatory responses to high-fat diet between C57BL6/J and C57BLKS/J inbred mouse strains. *Am. J. Physiol. Endocrinol. Metab.* 305, E1495-E1511. 10.1152/ajpendo.00366.2013 24169046 PMC3882376

[DMM028530C35] Sims-Robinson C., Bakeman A., Bruno E., Jackson S., Glasser R., Murphy G. G. and Feldman E. L. (2016). Dietary reversal ameliorates short- and long-term memory deficits induced by high-fat diet early in life. *PLoS ONE* 11, e0163883 10.1371/journal.pone.0163883 27676071 PMC5038939

[DMM028530C36] Singleton J. R., Marcus R. L., Lessard M. K., Jackson J. E. and Smith A. G. (2015a). Supervised exercise improves cutaneous reinnervation capacity in metabolic syndrome patients. *Ann. Neurol.* 77, 146-153. 10.1002/ana.24310 25388934 PMC4293306

[DMM028530C37] Singleton J. R., Smith A. G. and Marcus R. L. (2015b). Exercise as therapy for diabetic and prediabetic neuropathy. *Curr. Diab Rep.* 15, 120 10.1007/s11892-015-0682-6 26538074

[DMM028530C38] Smith A. G. and Singleton J. R. (2012). Diabetic neuropathy. *Continuum (Minneap Minn)* 18, 60-84. 10.1212/01.con.0000411568.34085.3e 22810070

[DMM028530C39] Smith A. G. and Singleton J. R. (2013). Obesity and hyperlipidemia are risk factors for early diabetic neuropathy. *J. Diabetes Complications* 27, 436-442. 10.1016/j.jdiacomp.2013.04.003 23731827 PMC3766404

[DMM028530C40] Smith A. G., Russell J., Feldman E. L., Goldstein J., Peltier A., Smith S., Hamwi J., Pollari D., Bixby B., Howard J. et al. (2006). Lifestyle intervention for pre-diabetic neuropathy. *Diabetes Care* 29, 1294-1299. 10.2337/dc06-0224 16732011

[DMM028530C41] Tabák A. G., Herder C., Rathmann W., Brunner E. J. and Kivimäki M. (2012). Prediabetes: a high-risk state for diabetes development. *Lancet* 379, 2279-2290. 10.1016/S0140-6736(12)60283-9 22683128 PMC3891203

[DMM028530C42] Tesfaye S., Boulton A. J. M., Dyck P. J., Freeman R., Horowitz M., Kempler P., Lauria G., Malik R. A., Spallone V., Vinik A. et al. (2010). Diabetic neuropathies: update on definitions, diagnostic criteria, estimation of severity, and treatments. *Diabetes Care* 33, 2285-2293. 10.2337/dc10-1303 20876709 PMC2945176

[DMM028530C43] Vincent A. M., Hayes J. M., Mclean L. L., Vivekanandan-Giri A., Pennathur S. and Feldman E. L. (2009). Dyslipidemia-induced neuropathy in mice: the role of oxLDL/LOX-1. *Diabetes* 58, 2376-2385. 10.2337/db09-0047 19592619 PMC2750230

[DMM028530C44] Wang L., Chopp M., Szalad A., Liu Z., Bolz M., Ãlvarez F. M., Lu M., Zhang L., Cui Y., Zhang R. L. et al. (2011). Phosphodiesterase-5 is a therapeutic target for peripheral neuropathy in diabetic mice. *Neuroscience* 193, 399-410. 10.1016/j.neuroscience.2011.07.039 21820491 PMC3391742

[DMM028530C45] Wiggin T. D., Sullivan K. A., Pop-Busui R., Amato A., Sima A. A. F. and Feldman E. L. (2009). elevated triglycerides correlate with progression of diabetic neuropathy. *Diabetes* 58, 1634-1640. 10.2337/db08-1771 19411614 PMC2699859

[DMM028530C46] Yorek M. S., Obrosov A., Shevalye H., Holmes A., Harper M. M., Kardon R. H. and Yorek M. A. (2015). Effect of diet-induced obesity or type 1 or type 2 diabetes on corneal nerves and peripheral neuropathy in C57Bl/6J mice. *J. Peripher. Nerv. Syst.* 20, 24-31. 10.1111/jns.12111 25858759 PMC4470853

